# Timely Delivery of Discharge Medications to Patients’ Bedsides: A Patient-centered Quality Improvement Project

**DOI:** 10.1097/pq9.0000000000000297

**Published:** 2020-05-08

**Authors:** Daphna T. Katz, Josaura V. Fernandez-Sanchez, Leah A. Loeffler, Simone M. Chang, Mora V. Puertolas-Lopez, Faizal R. Ramdial, Gabrielle R. Fisher, Susan A. Gutierrez, Neha Mahajan, Divya R. Keerthy, Stephania P. Cavallaro, Claudia E. Landaeta, Akilah S. Pascall, Kristina T. Acevedo, Kwai T. Chan-Poon, Benjamin R. Abraham, Matthew Siri, Kimberly L. Reynolds, Kendra Van Kirk, Liz Y. Bayes Santos

**Affiliations:** From the * Department of Pediatrics, Jackson Memorial Hospital; † University of Miami Miller School of Medicine, Miami, FL.

## Abstract

**Introduction:**

Patients who are unable to fill prescriptions after discharge are at risk of hospital readmission. Ensuring that patients have prescriptions in hand at the time of discharge is a critical component of a safe and effective discharge process. Using a “Meds to Beds” program, we aimed to increase the percentage of patients discharged from Holtz Children’s Hospital with medications in hand from 49% to 80%, reduce turnaround time (TAT) from electronic prescription signature to bedside delivery from 4.9 hours (±2.6 hours) to 2 hours, and increase caregiver satisfaction.

**Methods:**

We formed a multidisciplinary team and implemented 4 patient-centered interventions through iterative plan-do-study-act cycles. Statistical process control charts were used to understand the impact of the interventions over 10 months. Hospital length of stay and discharges before 2:00 pm were used as balancing measures. We measured caregiver satisfaction using a telephone survey administered by pediatric residents within 7 days after discharge.

**Results:**

The mean percentage of patients discharged with medications in hand increased to 76%. TAT decreased to 3.5 hours (±1.8 hours). Length of stay did not significantly increase, whereas the percentage of patients discharged before 2:00 pm did. Caregivers of patients who had prescriptions delivered to their bedside reported high levels of satisfaction.

**Conclusions:**

Using a “Meds to Beds” program, we increased the percentage of patients discharged with medications in hand, decreased TAT with reduced variability, and achieved high levels of caregiver satisfaction. Importantly, there was a shift in the culture of the institution toward improved medication access for patients.

## INTRODUCTION

The inability to obtain prescriptions following hospital discharge and the lack of knowledge regarding accurate medication administration can lead to reutilization of the healthcare system and medication errors.^1–5^ Known barriers that prevent patients from filling prescriptions include lack of transportation, issues with insurance companies, and unaffordable copays.^6^ Helping patients fill their prescriptions before discharge decreases hospital readmission rates.^7,8^ Review of medications by healthcare providers with patients at the bedside is also associated with improved long-term outcomes.^9,10^ Ensuring that patients have prescriptions in hand at the time of discharge is, therefore, a critical component of a safe and effective discharge process.^11,12^

Among patients discharged from the general pediatric unit at Holtz Children’s Hospital from September 2017 to December 2017, only 49% filled their prescriptions at an onsite outpatient pharmacy; the remainder were filled at other pharmacies or not at all. Some patients were made aware by the primary team of the option to have their medications delivered to the bedside by a pharmacy technician, and the patients requested this service. The other patients waited at the pharmacy to pick up their prescriptions. The average wait time for bedside delivery was 4.9 hours (±2.6 hours). This delay and the considerable variability in wait time were significant obstacles for pediatric providers attempting to discharge patients efficiently.

Mallory et al^11^ increased the percentage of pediatric patients leaving their hospital with new prescriptions in hand from 2% to 85% over 18 months by creating a bedside delivery service for medications from their onsite outpatient pharmacy. Given these results, we believed that by improving the existing option for bedside delivery of prescriptions from our onsite outpatient pharmacies, a program that has since been renamed “Meds to Beds,” we could increase the percentage of pediatric patients discharged with medications in hand.

The global aim of this project was to improve our discharge prescription process by increasing the percentage of patients leaving our hospital with all medications in hand to 80%. Our secondary aims were to decrease the turnaround time (TAT) from electronic prescription signature to bedside delivery to 2 hours and improve caregiver satisfaction over the ensuing 10 months.

## METHODS

### Context

This project was carried out at Holtz Children’s Hospital, a 217-bed facility located within Jackson Memorial Hospital (JMH), an academic center in Miami and the largest public hospital in the United States with 795 licensed beds. The inpatient pediatric unit, including general and subspecialty patients, has 60 beds, excluding newborn nursery, Neonatal Intensive Care Unit (NICU), and Pediatric Intensive Care Unit (PICU). The hospital primarily serves residents of Miami-Dade County, of which 76% are English speaking, 71.5% have Medicaid coverage, and 1% receive charity care from JMH in the form of a Jackson card, a Jackson Health System financial assistance program.

There are 2 onsite outpatient pharmacies; both offer a service for prescriptions to be delivered by 1 of 2 pharmacy technicians, directly to patients’ bedsides before discharge. A caregiver must be present at the bedside to accept pediatric prescriptions.

### Improvement Team

**Fig. 1.**
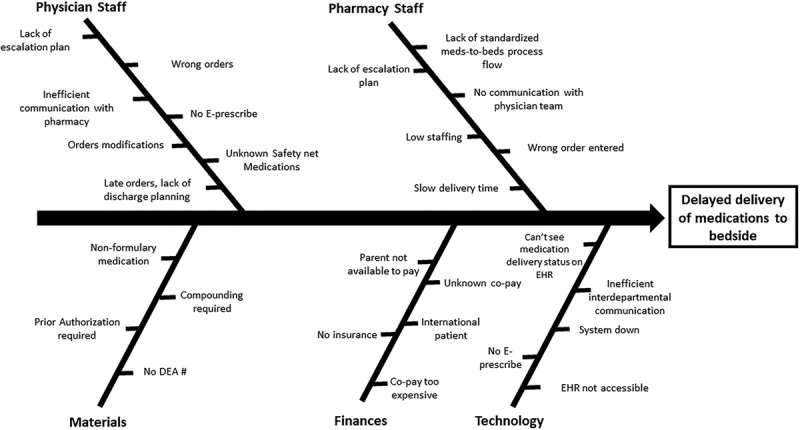
Ishikawa diagram (root cause analysis). DEA, Drug Enforcement Administration; SMART, Specific Measurable Attainable Realistic Timely.

In September 2017, we formed a team including first-, second-, and third-year pediatric residents, a Transitions of Care (ToC) inpatient clinical pharmacist whose role includes communication with the outpatient pharmacies, and a pediatric hospitalist. We held multidisciplinary meetings with nurses, social workers, case managers, and a quality improvement expert. A process map was created, and root cause analysis (Fig. 1) was performed to identify factors contributing to delays in the delivery of medications.

### Planning the Interventions

**Fig. 2.**
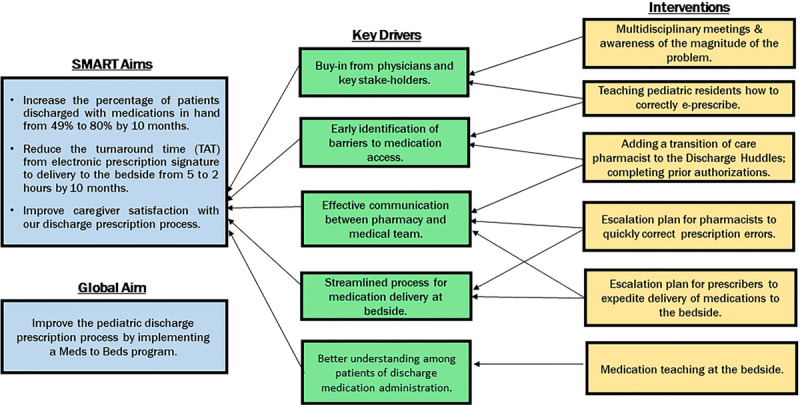
Key driver diagram.

We obtained baseline data from September through December 2017 from the electronic health records (EHRs; 2011 Cerner Corporation, Kansas City, Mo.), from which we generated a random sample of 20 patients per month over the 4 months using a stratified sampling methodology. Based on this information, we defined 3 measurable and time-bound specific aims. We created a Key Driver Diagram (Fig. 2) to guide our process of improvement. Using iterative plan-do-study-act (PDSA) cycles, we implemented patient-centered interventions theorized to have the most significant impact on these drivers.

### Interventions

From January to October 2018, we implemented 4 interventions. The first intervention was to add our ToC pharmacist from our Improvement Team to the “Discharge Huddle,” a multidisciplinary meeting held every weekday to discuss the anticipated discharge needs of all patients admitted to our general pediatric unit. Our rationale for this intervention was to create a direct channel for communication between the primary team and pharmacists to discuss discharge prescriptions and coordinate the delivery of prescriptions. Moreover, the ToC pharmacist reviews each patients’ insurance information and helps solve financial and logistical issues early in the admission, such as the need to obtain prior authorization for a medication.

The second intervention was to teach pediatric residents how to prescribe medications electronically to the hospital pharmacies and efficiently utilize the delivery service. For example, residents were instructed to indicate patients’ anticipated date of discharge in the prescription comments to coordinate the delivery time with the pharmacy. We created a 1-page reference sheet with relevant information for prescribers, such as the pharmacies’ phone numbers and hours of operation. An escalation plan was also included for providers to know who to contact in the case of delays in the delivery of medications. The sheet was presented at multiple program-wide meetings, displayed in the pediatric residents’ workroom, and made accessible to the pediatric residents by a smartphone application.

The third intervention was to improve communication between pharmacists and prescribers. We created a 1-page reference sheet for pharmacists that includes instructions on how to notify the appropriate pediatric resident of prescription errors quickly. The plan also included how to escalate to hospitalist attendings if initial attempts to contact pediatric residents were unsuccessful. We distributed and reviewed the reference sheet with the outpatient pharmacists and posted it in the outpatient pharmacies.

The final intervention provided reminders to pediatric residents to continue correctly prescribing to an onsite pharmacy (rather than to an outside pharmacy), utilize the prescription bedside delivery service, and effectively communicate with pharmacy staff. For example, we asked that all pediatric hospitalist attendings notify senior pediatric residents of the expectation to adhere to the project goals, and the chief residents provided a short training to pediatric interns at the start of each rotation. We taped small notecard reminders to pediatric resident computer workstations on wheels.

### Study of the Interventions

One pediatric resident was assigned weekly to review the electronic charts of all inpatients discharged on weekdays from our general pediatrics unit. We did not include the subspecialty teams due to the greater complexity of their discharge prescriptions. Weekend data were not collected due to limited pharmacy hours when compared with weekdays. Additionally, the Discharge Huddle meets only on weekdays. Information regarding patients’ discharge prescriptions was input into a Redcap database. The ToC pharmacist added the times that prescriptions were delivered to patients’ bedsides, as documented by the pharmacy technicians in their delivery records.

The assigned pediatric resident conducted a telephone survey to caregivers of patients discharged the previous day on 3 weekdays during working hours. The resident made 3 attempts to contact a caregiver within 7 days after hospital discharge. Caregivers were asked how satisfied they were with the teaching provided about their child’s medication(s) and with the overall discharge medication process. Non-English-speaking caregivers were contacted using a licensed translator phone service.

### Measures and Analysis

We defined the outcome measure as the percentage of patients discharged with prescriptions in hand; we calculated this measure every 2 weeks by dividing the number of patients who had prescriptions delivered to the bedside by the total number of patients who required prescriptions at the time of discharge.

The process measure was TAT, which we calculated by subtracting the time that the pediatric resident electronically signed each prescription from the time recorded by the technician that they delivered the prescription to the bedside. When the pharmacy was closed, TAT was calculated by subtracting the pharmacy opening hour on the day of delivery from the time recorded by the technician that they delivered the prescription to the bedside. We used X-bar and S control charts to display the mean and SD of the TAT biweekly, respectively.

The final measure was caregiver satisfaction, as measured by the results of the telephone survey administered by pediatric residents. Options included very satisfied/satisfied/dissatisfied/very dissatisfied.

Our balancing measures were mean hospital length of stay (LOS) and the percentage of patients discharged from the hospital before 2:00 pm, the hospital’s metric for timely discharges. These data were determined by reviewing the time when admission and discharge orders were placed in the EHR.

Statistical process control and run charts displayed the biweekly percentage of patients with medications delivered to their bedside, mean TAT, mean LOS, and the percentage of patients discharged before 2:00 pm. Data over time were used to monitor and understand the impact of changes in the discharge prescription process. We established a mean, illustrated as the centerline on all control charts with upper and lower control limits. We used the Montgomery rules to determine if observed changes in measures were due to random variation (common cause variation) or caused by a specific assignable cause, in this case, the intervention (special cause variation).^13^ We also used the Fisher test and Wilcoxon rank-sum to calculate *P* values to determine statistical significance between the baseline and intervention period according to Lean Methodology using SAS version 9.4 (SAS Institute, Inc, Cary, N.C.).

### Ethical Considerations

The present initiative fell within the JMH/University of Miami Institutional Review Board’s guidance for quality improvement projects that did not constitute human subjects research. We did not obtain external funding.

## RESULTS

Baseline data revealed that 58.8% of patients (n = 47) admitted in September to December 2017 required prescriptions at the time of discharge. During that time, 49% of those patients (n = 23) filled their prescriptions at one of the onsite pharmacies before discharge. Among patients who requested delivery to the bedside, the average wait time was 4.9 hours (± 2.6 hours). Additional baseline information showed that the hospital discharged 28.8% of patients (n = 23) before 2:00 pm, and the average LOS was 2.8 days (±3.2 days).

**Fig. 3.**
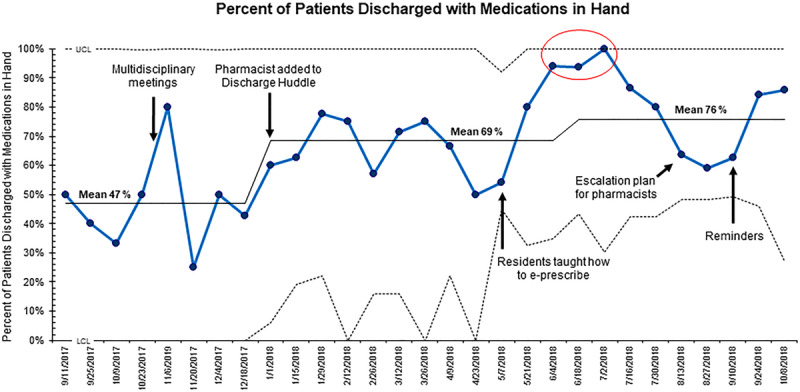
Statistical process control (p-chart) displaying the biweekly percentage of patients discharged with medications in hand. A baseline of 47% is observed. After 4 PDSA cycles, the mean went up to 76%. LCL indicates lower control limit (3 SDs below the mean); UCL, upper control limit (3 SDs above the mean).

Similar to the baseline data, during the intervention period, 59.1% of the total patients (n = 272) admitted required prescriptions at the time of discharge. Following the first intervention, which involved adding a ToC pharmacist to the Discharge Huddle, the mean percentage of patients discharged with medications in hand increased to 69%. With the subsequent interventions, the mean centerline increased to 76% (Fig. 3). In total, 71.7% of the patients (n = 195) who required prescriptions at the time of discharge during the project implementation period went home with medications in hand. This improvement was a statistically significant increase (*P* = 0.0024) from the baseline total of 48.9%.

**Fig. 4.**
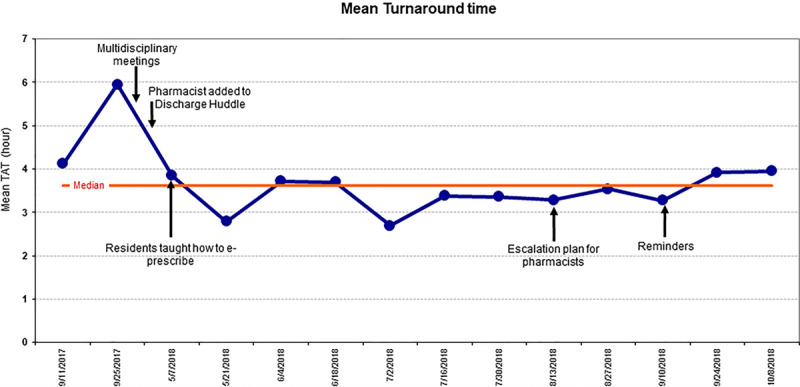
Run chart of the mean TAT defined as the time from prescription signature to prescription delivery to the bedside. Median TAT is 3.6 hours.

Concerning the process measure, there was a decrease in TAT from a baseline of 4.9 hours (±2.6 hours) to 3.5 hours (±1.8 hours) (n = 144), which was a statistically significant decrease (*P* = 0.0021). However, we could not identify any rule of special cause variation in the run chart (Fig. 4).

**Fig. 5.**
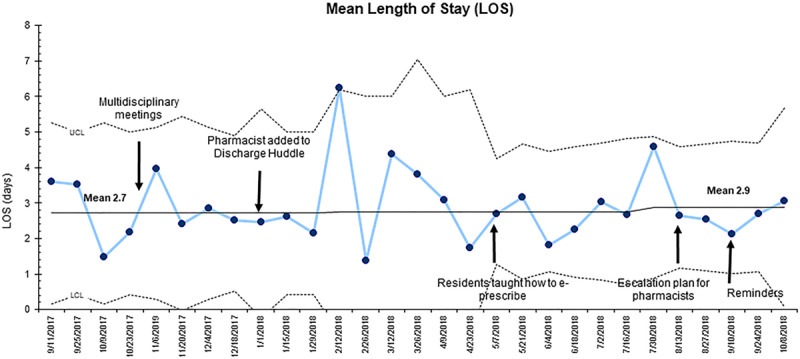
Statistical process control (X-chart) measuring the mean LOS. An increase from 2.7 to 2.9 days was observed. LCL indicates lower control limit (3 SDs below the mean); UCL, upper control limit (3 SDs above the mean).

**Fig. 6.**
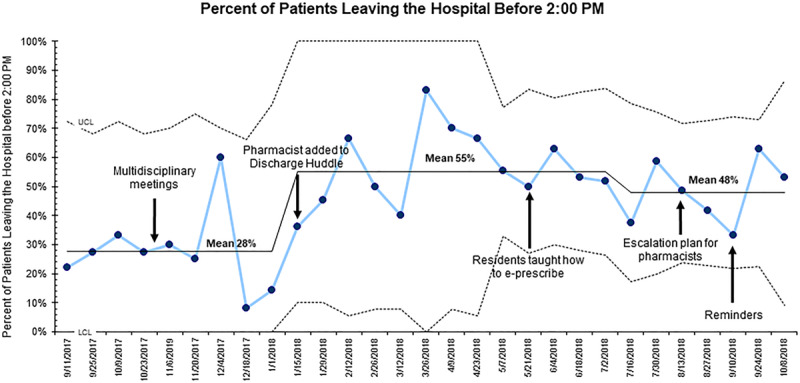
Statistical process control (p-chart) displaying the biweekly percentage of patients being discharged from the hospital before 2:00 pm. A baseline of 28% is observed. After 4 PDSA cycles, the mean went up to 48%. LCL indicates lower control limit (3 SDs below the mean); UCL, upper control limit (3 SDs above the mean).

A review of balancing measures showed that there was no statistically significant difference in hospital LOS, as following the interventions, the mean was 2.8 days (±4.2 days) compared with 2.8 days (±3.2 days) (*P* = 0.91) (Fig. 5). However, there was a shift in the mean centerline from 2.7 to 2.9 days. The percentage of patients discharged before 2:00 pm increased from a mean of 28% to 48% following the implementation period (Fig. 6).

We obtained follow-up caregiver satisfaction surveys via telephone for 63 patients during the intervention period. Of those patients, 92% (n = 58) had medications delivered to their bedside. In terms of caregiver satisfaction, 96% of caregivers of patients (n = 53) whose prescriptions were delivered to their bedside reported the highest level of satisfaction with the teaching they received regarding their medications. Similarly, 96% of caregivers of patients (n = 54) whose prescriptions were delivered to their bedside reported the highest level of satisfaction with the overall discharge prescription process.

## DISCUSSION

The global aim of this project was to improve our discharge prescription process to increase patient possession of medications needed at the time of discharge. This venture was particularly important, given the low socioeconomic status of our complex patient population, many of whom are likely to encounter barriers to filling prescriptions after they have been discharged. The main strategy utilized was to improve our existing “Meds to Beds” program. The project was successful in increasing the percentage of patients going home with prescriptions in hand without negatively impacting balancing measures significantly.

### Primary Outcome

The most effective intervention appeared to be the addition of our ToC pharmacist to the Discharge Huddle, likely given the lack of and apparent strong need for effective communication between pharmacists and prescribers. Durkin et al^14^ and Hatoun et al^7^ both implemented delivery services for medications in their emergency room and inpatient units, respectively, that similarly relied on a partnership between pharmacists and physicians for successful implementation. Teaching pediatric residents to plan for discharge early in patients’ hospital admission was also effective and has proven to be so at multiple institutions.^15,16^

Though the interventions did not result in our goal of discharging 80% of patients with medications in hand, we observed a significant increase from baseline. An example in the literature in which a higher percentage was achieved involved delivery of only new prescriptions to patients’ bedsides, whereas we aimed to discharge patients with all of their medications, a more challenging goal given some caregiver preferences for an external pharmacy. Moreover, we were not able to ensure that all pediatric residents participated in “Meds to Beds.”

Overall, we raised awareness about the importance of improved medication access for patients at our hospital. It is now standard practice to discuss the need for “Meds to Beds” during pediatric general team rounds, and the ToC pharmacist continues to be present at all Discharge Huddles. Moreover, in light of our project, in April 2019, a “Meds to Bed” order was added to the Pediatric Discharge Power Plan in our EHR.

### Process and Balancing Measures

We achieved a decrease in 1.5 hours from the baseline TAT using traditional statistical analysis. This decrease was likely a result of training pediatric residents on electronic prescribing and improved communication between pharmacists and prescribers. Importantly, the variability in waiting time decreased as evidenced by the reduced SD; this will allow pediatric residents to predict more accurately when prescriptions must be signed electronically so as not to delay patients’ anticipated discharge time. Though we did not achieve a TAT of 2 hours, this may not have been a realistic goal given our high patient volume and the currently limited number of available pharmacy technicians, as well as the need for caregivers to be present to accept pediatric prescriptions. We were also likely not able to achieve special cause variation, due to our limited data on TAT.

Though we did see an increase in the centerline for mean LOS following our interventions, this was not a statistically significant increase, a potential complication of waiting for delivery of medications. This finding was consistent with the study done by Hatoun et al^7^ in which the mean LOS did not significantly deviate from the baseline of 2.6 days between PDSA cycles.

We also did not negatively impact the percentage of patients discharged before 2:00 pm. Instead, there was an increase, which may have been due in part to the focus on efficient hospital discharge planning. The administration at Holtz Children's Hospital launched an initiative that coincided with the implementation period to increase the number of patients discharged before 2:00 pm, which likely positively impacted these results.

### Caregiver Satisfaction

Caregiver satisfaction is frequently used as an important quality measure for pediatric hospital discharges.^7^ This project’s goal was to improve satisfaction with the overall discharge process by increasing the number of patients going home with medications in hand. The vast majority of caregivers surveyed reported the highest level of satisfaction with the teaching they received regarding their medications as well as the overall discharge process when we delivered their prescriptions to the bedside. This result is consistent with the study by Mallory et al,^11^ who showed an increase in caregiver satisfaction from 50% to 88% after the introduction of their bedside delivery program.

Improved satisfaction likely stems from healthcare providers, including physicians and nurses, educating caregivers about their medications at the bedside. This process cannot be guaranteed when prescriptions are picked up from the pharmacy. Moreover, caregivers seem to enjoy the convenience of the delivery service.^17^

### Limitations

We were limited by the fact that we collected data over a relatively short study period of 10 months. This time was likely not sufficient to fully demonstrate the effectiveness of our interventions. We also had a small pool of data on TAT, which likely impacted our ability to demonstrate special cause variation. This limitation is because our onsite pharmacies previously documented medication delivery times using hand-written logs for the entire hospital that were difficult to interpret, given missing pages, and being combined with adult data. Data collection of TAT improved in May 2018 during our second PDSA cycle, when technicians began electronic recording on an iPad. Furthermore, the process of obtaining caregiver satisfaction surveys was very time-consuming for pediatric residents, mainly due to many caregivers not answering their phones or leaving an inaccurate phone number in their child’s chart. We also had no baseline satisfaction data to assess the impact of our interventions on caregiver satisfaction fully.

### Future Directions

Future directions will include continuing the current interventions and formally applying them to the subspecialty teams that are already increasingly utilizing “Meds to Beds.” We also hope to standardize our medication teaching at the bedside.

## CONCLUDING SUMMARY

Using a “Meds to Beds” program, we improved the discharge prescription process at Holtz Children’s Hospital. The percentage of patients discharged with medications in hand greatly increased without significantly impacting hospital LOS, and caregivers were highly satisfied. Importantly, there was a shift in the overall culture of the institution toward improved medication access for patients.

## DISCLOSURE

The authors have no financial interest to declare in relation to the content of this article.

## ACKNOWLEDGMENTS

We thank Jasmine Nebhrajani, DO, Prashant Minocha, MD, Daniela Aguilar-Caballero, MD, Juan Palacio, MD, Sofia Saenz-Ayala, MD, Josef Newman, MD, Alicia Morrison, MD, Patricia Arroyo Parejo, MD, Paulo Nino, MD, Chetan Nanjegowda, MD, and Emily Stumpf, DO for their assistance with the study and the data collection. The authors thank the pharmacy staff and nurses at Holtz Children’s Hospital for their cooperation during this project.
